# Simultaneous Analysis of Paracetamol and Diclofenac Using MWCNTs-COOH Modified Screen-Printed Carbon Electrode and Pulsed Potential Accumulation

**DOI:** 10.3390/ma13143091

**Published:** 2020-07-10

**Authors:** Agnieszka Sasal, Katarzyna Tyszczuk-Rotko, Magdalena Wójciak, Ireneusz Sowa, Michał Kuryło

**Affiliations:** 1 Faculty of Chemistry, Institute of Chemical Sciences, Maria Curie-Skłodowska University in Lublin, 20-031 Lublin, Poland; agnieszkaszwagierek@gmail.com; 2 Department of Analytical Chemistry, Medical University of Lublin, 20-093 Lublin, Poland; kosiorma@wp.pl; 3 Municipal Water Supply & Waste Water Treatment Company Ltd., Central Laboratory, 20-245 Lublin, Poland; michal.kurylo@wp.pl

**Keywords:** carboxyl functionalized multiwalled carbon nanotubes modified screen-printed carbon electrode, paracetamol and diclofenac, pulsed potential accumulation, voltammetry, environmental water and sewage samples, direct analysis, liquid chromatography

## Abstract

A differential-pulse adsorptive stripping voltammetric (DPAdSV) procedure with the use of pulsed potential accumulation and carboxyl functionalized multiwalled carbon nanotubes modified screen-printed carbon electrode (SPCE/MWCNTs-COOH) was delineated for simultaneous analysis of paracetamol (PA) and diclofenac (DF). The use of carboxyl functionalized MWCNTs and pulsed potential accumulation improves the analytical signals of PA and DF, and minimizes interferences from surfactants. After optimization of analytical conditions for this sensor, the peak currents of the two compounds were found to increase linearly with the increase in their concentration (5.0 × 10^−9^–5.0 × 10^−6^ mol L^−1^ with a detection limit of 1.4 × 10^−9^ mol L^−1^ for PA, and 1.0 × 10^−10^–2.0 × 10^−8^ mol L^−1^ with a detection limit of 3.0 × 10^−11^ mol L^−1^ for DF). For the first time, the electrochemical sensor allows simultaneous determination of PA and DF at concentrations of 24.3 ± 0.5 nmol L^−1^ and 3.7 ± 0.7 nmol L^−1^, respectively, in wastewater samples purified in a sewage treatment plant.

## 1. Introduction

Paracetamol (PA) is a very popular drug with an antipyretic effect, caused by inhibition of prostaglandin synthesis in the central nervous system. PA has potent antipyretic and analgesic effects, but no anti-inflammatory effect. Indications for administration of the drug include fever and acute and chronic pain. PA is recommended by the World Health Organization as one of the basic drugs in the treatment of pain during cancer. In addition, it is used for headaches, including migraine, earaches, toothaches, menstruation, and neuralgia, as well as rheumatic, myofascial, bone, postoperative, and other pains [1,2].

Diclofenac (DF) belongs to the group of nonsteroidal anti-inflammatory drugs (NSAIDs). Thanks to its chemical structure, it is classified as a phenylacetic acid derivative. DF exhibits activities characteristic of the NSAID group, that is, anti-inflammatory, analgesic, antipyretic, and inhibiting platelet aggregation. The basis of the mechanism of action is inhibition of cyclooxygenase, an enzyme involved in the synthesis of prostaglandins from cell membrane lipids. DF is used to treat inflammation and rheumatic (including rheumatoid arthritis) and non-rheumatic pain (including postoperative and traumatic pains, gout attacks, renal and hepatic colic, and dysmenorrhea) [3,4].

The constantly growing production of medicines adversely affects the natural environment, primarily polluting water reservoirs. As studies show [5,6], after leaving a sewage treatment plant, the water still contains numerous active substances of pharmaceutical preparations, which then end up in the natural environment. The presence of commonly used pharmaceuticals in water ecosystems poses a threat to fish and other water organisms, as well as for human and animal health. DF and PA are some of the most commonly found drugs in environmental water samples and their concentrations are about 10^−11^–10^−8^ and 10^−9^–10^−8^ mol L^−1^, respectively [7,8].

There are many methods in the literature describing the simultaneous analysis of PA and DF. These are chromatographic methods based on high performance liquid chromatography [9,10,11,12] and gas chromatography with mass spectroscopy [13], as well as spectrophotometry [14] and electrophoresis [15,16]. However, these methods usually require a time- and reagent-consuming step of sample preparation process for determination of low concentration of PA and DF in samples.

Electrochemical methods, which are cheap, simple, fast, and environmentally friendly because they consume very small amounts of chemical reagents, are an alternative to these methods. According to the best of our knowledge, in the literature, there are only three works about the application of voltammetric sensors for the simultaneous determination of PA and DF, which are based on the use of glassy-carbon electrodes modified with 4-phosphatephenyl [17] or polymer functionalized graphene [18,19]. Only one of them [18] shows the use of an electrochemical sensor for the simultaneous determination of PA and DF in water samples. Unfortunately, PA and DF were determined in spiked lake water samples at concentrations (around 10^−5^ mol L^−1^) much higher than those actually present in environmental samples given that the obtained values for the limit of detection of PA and DF were 2.2 × 10^−7^ and 6.1 × 10^−7^ mol L^−1^, respectively.

Electrochemical sensors based on screen-printing technology are a good solution for quick and routine tests both in the laboratory and directly in the environment. Screen-printed electrodes are cheap and ready-made systems with various modifications, and they are easily available commercially. Purchased electrochemical sensors do not require additional modifications; are immediately ready for use; and are characterized by high selectivity, sensitivity, and reproducibility [2,4].

The sales dynamics of pharmaceuticals confirm the growing problem of contamination of the water environment. This makes monitoring the water environment for the presence and content of residues of pharmaceuticals a significant issue for contemporary analytical chemistry. The goal of this work was to show the voltammetric procedure for the simultaneous analysis of a low concentration of DF and PA in environmental water and sewage samples using a screen-printed sensor without the sample pre-treatment step. Additionally, for the first time, in order to improve PA and DF analytical signals as well as to minimize interferences from surfactants, pulsed potential accumulation was applied.

## 2. Materials and Methods

### 2.1. Instrumentations

Cyclic voltammetric (CV) and differential-pulse adsorptive stripping voltammetric (DPAdSV) studies were carried using an electrochemical analyzer (µAutolab, Eco Chemie, Utrecht, Netherland) managed by GPES 4.9 software. All electrochemical experiments were performed in a 10 mL classic cell with commercially available carboxyl functionalized multiwalled carbon nanotubes modified screen-printed carbon electrodes (SPCE/MWCNTs-COOH, DropSens, Llanera, Spain, Ref. 110CNT). This three-electrode system contained of a screen-printed carbon electrode covered by carboxyl functionalized multiwalled carbon nanotubes (working electrode with a diameter of 4 mm), SPCE (auxiliary electrode), and a screen-printed silver electrode (pseudo-reference electrode). The results at the SPCE/MWCNTs-COOH were compared to those obtained using a commercially available screen-printed carbon electrode (SPCE, DropSens, Llanera, Spain, Ref. C110) and a commercially available screen-printed carbon/carbon nanofibers electrode (SPCE/CNFs, DropSens, Llanera, Spain, Ref. 110CNF).

The microscopic images of the SPCE/MWCNTs-COOH sensor surface were recorded using an optical microscope and a high-resolution scanning electron microscope Quanta 3D FEG (FEI, Hillsboro, OR, USA).

VWR Hitachi Elite LaChrom HPLC system equipped with a spectrophotometric detector (PAD) and EZChrom Elite software (version 3.3.2 SP2, Merck, Darmstadt, Germany) was used for chromatographic analysis. The XB-C18 reversed phase core-shell column (Kinetex, Phenomenex, Aschaffenburg, Germany) (25 cm × 4.6 mm i.d., 5 μm particle size) was used in HPLC-PAD measurements.

### 2.2. Chemicals

The reagents purchased from the company Sigma-Aldrich (Saint Louis, MO, USA), paracetamol sulfate potassium salt (PA) and 2-[(2,6-dichlorophenyl)amino]benzeneacetic acid sodium salt (DF), were dissolved in distilled water to prepare 0.01 mol L^−1^ solutions of PA and DF, respectively. According to needs, these solutions were diluted using distilled water. During the tests, the following supporting electrolyte solutions were used: acetic acid, acetate buffer (CH_3_COONa + CH_3_COOH) with pH values of 3.4 ± 0.1, 3.8 ± 0.1, 4.0 ± 0.1, 4.4 ± 0.1, 5.0 ± 0.1, 5.4 ± 0.1, and 6.0 ± 0.1, prepared from Sigma-Aldrich reagents. The standard solutions of uric acid, urea, ascorbic acid, glucose, and dopamine (Sigma-Aldrich, Saint Louis, MO, USA), as well as Cu^2+^, Fe^3+^, Cd^2+^, Mo^6+^, Ni^2+^, Pb^2+^, Zn^2+^, Sb^3+^, V^5+^, K^+^, Na^+^, Cl^−^, SO_4_^2−^, PO_4_^3−^, and NO_3_^−^ (Merck, Darmstadt, Germany), were used in interference studies. The influence of Triton X-100 was investigated based on a reagent obtained from Fluka (Charlotte, NC, USA). HPLC-grade acetonitrile and trifluoroacetic acids (TFAs) were purchased from Merck (Darmstadt, Germany). Ultrapurified water (>18 MΩ cm, Milli-Q system, Millipore, UK) was used for the preparation of solutions.

### 2.3. DPAdSV Procedure

**Figure 1:**
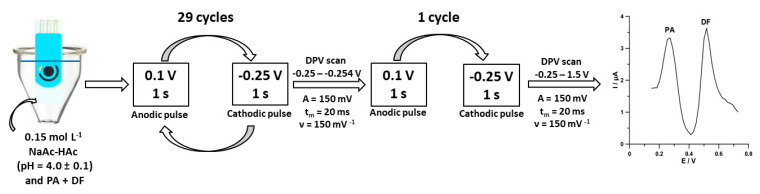
Scheme of voltammetric measurements of paracetamol (PA) and diclofenac (DF) at the SPCE/MWCNTs-COOH.

Under optimized conditions, differential-pulse adsorptive stripping voltammetric determinations of PA and DF were performed in 0.15 mol L^−1^ acetate buffer (pH of 4.0 ± 0.1) using pulsed potential accumulation (Figure 1). The procedure consisting of a 1 s accumulation period at a potential of 0.1 V (the anodic pulse) and a 1 s accumulation period at a potential of −0.25 V (the cathodic pulse) was repeated 30 times. The differential-pulse scans from −0.25 to −0.254 V with an amplitude (*A*) of 150 mV, a modulation time (*t_m_*) of 20 ms, and a scan rate (*ν*) of 150 mV s^−1^ were recorded after 29 accumulation cycles. In the last cycle, the differential-pulse scan from −0.25 to 1.5 V was recorded with the parameters described above.

### 2.4. HPLC/PAD Procedure

The chromatographic analysis was based on literature data [20] with a slight modification of the eluent composition. A mixture of acetonitrile and water with 0.025% of trifluoroacetic acid in proportion of 60:40 v/v for DF and 15:85 v/v for PA was used in analysis. The flow rate of the mobile phase was 1.0 mL min^−1^ and the temperature of the thermostat was set to 25 °C. Injection volumes were 80 µL. All samples were analysed at a wavelength of 276 nm for DF and 248 nm for PA 9 (n = 3).

### 2.5. Direct Analysis of Water Samples

The Bystrzyca river water samples (Lublin, Poland) and waste effluents purified in a sewage treatment plant (Lublin, Poland) were analyzed using the voltammetric and chromatographic methods. The samples were directly analyzed without sample pretreatment procedure.

## 3. Results and Discussion

### 3.1. Screen-Printed Electrode Selection and Surface Studies

**Figure 2:**
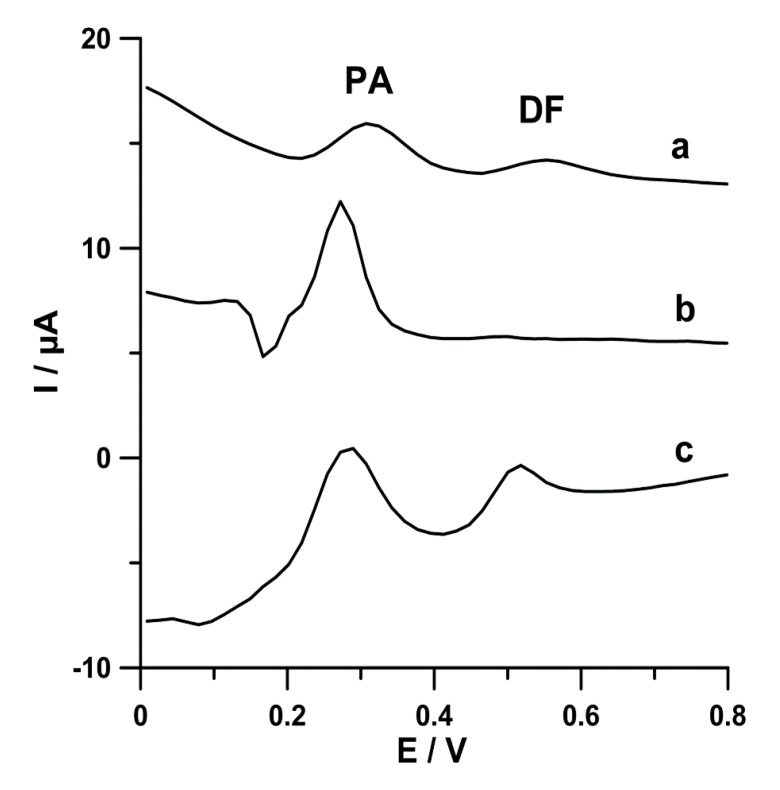
Differential-pulse adsorptive stripping voltammetric (DPAdSV) curves of PA (2.0 × 10^−6^ mol L^−1^) and DF (2.0 × 10^−8^ mol L^−1^) in 0.1 mol L^−1^ acetate buffer solution of pH 4.0 ± 0.1 at SPCE (a), carbon nanofibers modified SPCE (SPCE/CNFs) (b), and SPCE/MWCNTs-COOH (c). The DPAdSV parameters are as follows: *E_acc._* −0.25 V, *t_acc._* 60 s, *A* 125 mV, *t_m_* 10 ms, and *ν* 175 mV s^−1^.

In order to compare the PA (2.0 × 10^−6^ mol L^−1^) and DF (2.0 × 10^−8^ mol L^−1^) signals at commercially available screen-printed carbon sensors (screen-printed carbon electrode, SPCE; carboxyl functionalized multiwalled carbon nanotubes modified SPCE, SPCE/MWCNTs-COOH; carbon nanofibers modified SPCE, SPCE/CNFs), the differential-pulse adsorptive stripping voltammetric curves were registered (Figure 2). PA and DF were accumulated at a constant value of potential of −0.25 V (*E_acc._*) for 60 s (*t_acc._*). The results demonstrated the small peaks of PA (2.1 µA) and DF (1.0 µA) at the SPCE (curve a). When the surface of the working electrode was coated with carbon nanofibers (curve b), the PA peak current was grown to 5.8 µA, but the DF signal was ill-defined (0.12 µA). The CNTs blocked the active surface of electrode for the DF molecules. In the case of the SCPE modified with MWCNTs-COOH, two well-defined peaks of PA (5.0 µA) and DF (2.3 µA) are visible (curve c). Moreover, the lowest background current was obtained at the SPCE/MWCNTs-COOH. It is obvious that, in the case of simultaneous determination of PA and DF, the SPCE/MWCNTs-COOH should be chosen. However, for the individual PA determination, the SPCE/CNFs should be used. These results perfectly confirm our previous research already described in the literature [2,4]. In the next step of the experiments, attempts were made to explain these differences between the size of PA and DF signals at the electrodes.

**Figure 3:**
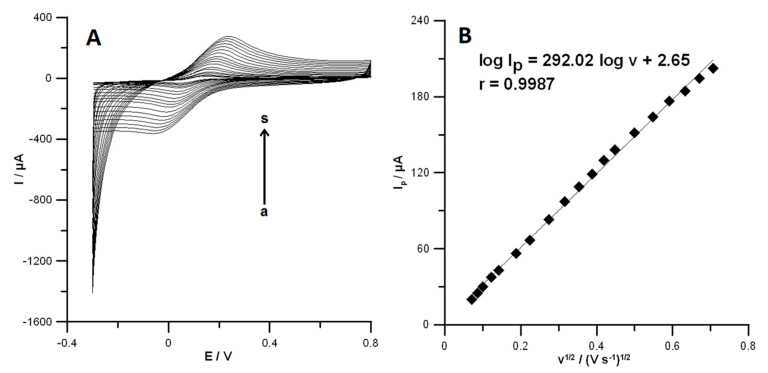
(**A**) Cyclic voltammetric (CV) curves obtained in solution containing 0.1 mol L^−1^ KCl and 5.0 × 10^−3^ mol L^−1^ K_3_[Fe(CN)_6_] at the SPCE/CNFs for the scan rate range from 5 to 500 mV s^−1^ (a–s). (**B**) The dependence between anodic peak current and scan rate square roots for SPCE/CNFs.

In the previously published papers [4], the electrochemical properties of SPCE and SPCE/MWCNTs-COOH were tested using CV studies in a solution of 0.1 mol L^−1^ KCl and 5.0 × 10^−3^ mol L^−1^ K_3_[Fe(CN)_6_]. However, the electrochemical properties SPCE/CNFs were not studied. Therefore, the active surface of SPCE/CNFs was examined using CV in a solution of 0.1 mol L^−1^ KCl and 5.0 × 10^−3^ mol L^−1^ K_3_[Fe(CN)_6_]. The cyclic voltammograms were recorded at different scan rates in the range from 5 to 500 mV s^−1^ (Figure 3A). The peak-to-peak separation (*ΔE*) for the SPCE/CNFs was estimated for the selected scan rate (175 mV s^−1^) as 169.0 ± 1.7 mV (n = 3). The results indicate the improvement of the reversibility process using CNFs-modified and especially MWCNT-COOH (*ΔE* = 149.0 ± 1.5 mV) electrodes in comparison with the unmodified SPCE (189.0 ± 1.9 mV) [4]. The dependence between anodic peak currents (*I_p_*) and square root of the scan rates (*v*^1/2^) was plotted (Figure 3B). On the basis of the Randles–Sevcik equation [21], the active surface area (*A_s_*) of the SPCE/CNFs was calculated. It should be mentioned that the geometric surfaces of all electrodes are the same. For the unmodified SPCE and SPCE/MWCNTs-COOH, the *A_s_* equals 0.061 ± 0.00058 cm^2^ (n = 3) and 0.10 ± 0.00097 cm^2^ (n = 3) [4], respectively, while the area of SPCE/CNFs was calculated to be 0.08090 ± 0.0014 cm^2^ (n = 3). The results show that the SPCE/MWCNTs-COOH has a greater number of active centers than the unmodified SPCE and the SPCE/CNFs. These results explain the enhancement of PA and DF signals in relation to the SPCE, and the DF signal in relation to the SPCE/CNFs. Moreover, DF may have a higher affinity to the SPCE/MWCNTs-COOH surface than SPCE/CNTs and SPCE owing to the surface functionalization with carboxyl (hydrophilic) groups. A slight difference in the peak current of PA at the SPCE/CNFs and SPCE/MWCNTs-COOH (5.8 µA vs. 5.0 µA, respectively) is owing to the fact that the SPCE/CNTs surface better facilitates the adsorption of PA. The electrochemical oxidation process of PA at the SPCE/CNFs surface is purely adsorption-controlled [2]. However, the goal of this work was to show the voltammetric procedure for the simultaneous analysis of DF and PA, and thus the SPCE/MWCNTs-COOH was chosen for further electrochemical study.

**Figure 4:**
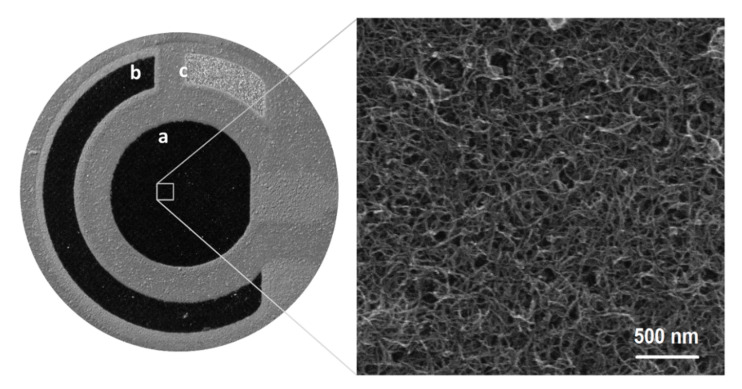
Optical (left side) and scanning electron microscopic (right side) images of SPCE/MWCNTs–COOH surface.

The selected three-electrode system surface consisting of an SPCE/MWCNTs–COOH (working electrode, a), an SPCE (auxiliary electrode, b), and an SPAgE (pseudo-reference electrode, c) was visualized by optical and scanning electron microscopes (Figure 4). It is apparent that the MWCNTs-COOH adheres to the carbon and is distributed homogeneously on the surface [4].

### 3.2. Effect of pH

**Figure 5:**
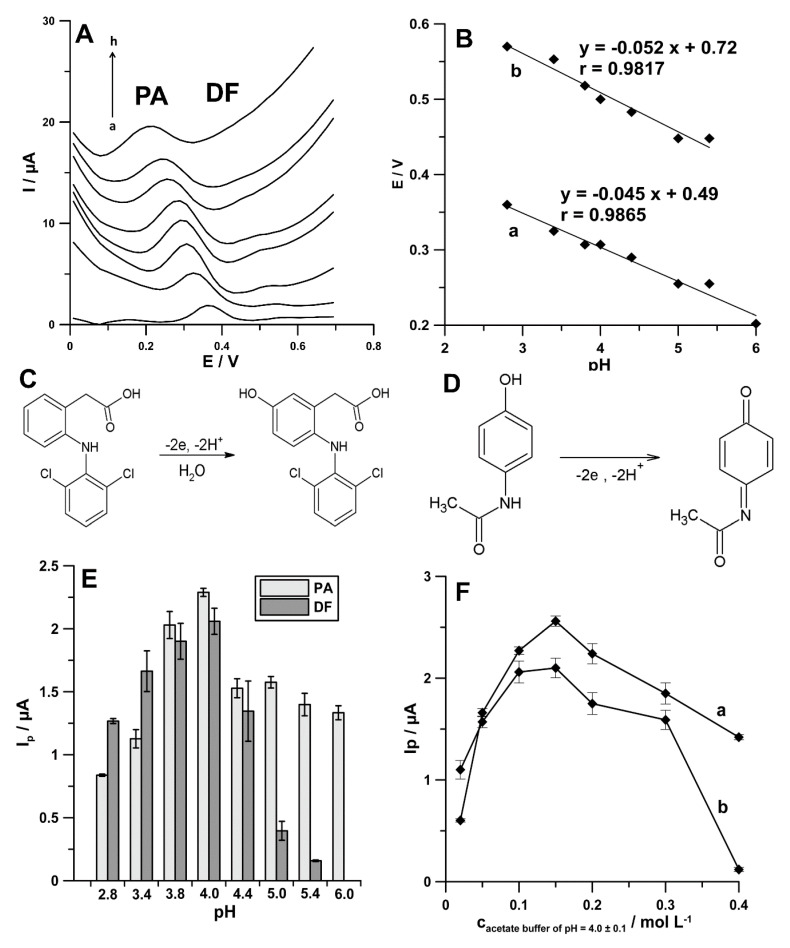
(**A**) DPAdSV curves recorded in 0.1 mol L^−1^ solutions of acetic acid (a), acetate buffer solution with pH values of 3.4 ± 0.1 (b), 3.8 ± 0.1 (c), 4.0 ± 0.1 (d), 4.4 ± 0.1 (e), 5.0 ± 0.1 (f), 5.4 ± 0.1 (g), and 6.0 ± 0.1 (h) containing PA (1.0 × 10^−6^ mol L^−1^) and DF (1.0 × 10^−9^ mol L^−1^). (**B**) The relationships between potential peaks of PA (a) and DF (b) and pH. Oxidation mechanisms of DF (**C**) and PA (**D**). Effect of different pH values (**E**) and the concentration of acetate buffer solution of pH 4.0 (**F**) on the 1.0 × 10^−6^ mol L^−1^ PA (a) and 1.0 × 10^−8^ mol L^−1^ DF (b) current responses.

The supporting electrolyte pH influences the peak potential and current as well as the shapes of the signals of biologically active compounds. Therefore, choosing an appropriate pH value is an important step during the optimization procedure. Here, 0.1 mol L^−1^ solutions of acetic acid and acetate buffer solutions with pH values of 3.4 ± 0.1, 3.8 ± 0.1, 4.0 ± 0.1, 4.4 ± 0.1, 5.0 ± 0.1, 5.4 ± 0.1, and 6.0 ± 0.1 containing PA (1.0 × 10^−6^ mol L^−1^) and DF (1.0 × 10^−9^ and 1.0 × 10^−8^ mol L^−1^) were examined. The results indicate that the potential peaks of PA and DF shifted to less positive values as pH increased (Figure 5A), indicating that protons were directly involved in the electrode reaction. Additionally, in Figure 5B, the relationships between potential peaks of PA and DF and pH are shown. As can be seen, the slopes of −45.0 mV pH^−1^ (for PA) and −52.0 mV pH^−1^ (for DF) were close to the theoretical value of −59.0 mV pH^−1^. These results indicate that the number of protons and transferred electrons involved in the oxidation mechanism of PA and DF is equal [21]. As PA and DF oxidation is a two-electron process the number of protons was also predicted to be 2, indicating the 2e^−^/2H^+^ process. DF is oxidized to 5-hydrohydiclofenac (Figure 5C) and PA to N-acetyl-p-quinoneimine (Figure 5D) [22,23].

Furthermore, it was observed that the peak current of PA and DF increased with increasing pH value to 4.0, and then the anodic peaks decreased (Figure 5E). Therefore, the acetate buffer solution of pH 4.0 was chosen as the supporting electrolyte in the simultaneous PA and DF determination. Moreover, it was found that the highest values of PA and DF signals were attained at 0.15 mol L^−1^ concentration of acetate buffer solution of pH 4.0, and hence it was further used (Figure 5F).

### 3.3. Accumulation of PA and DF at SPCE/MWCNTs-COOH and Sensor Selectivity

**Figure 6:**
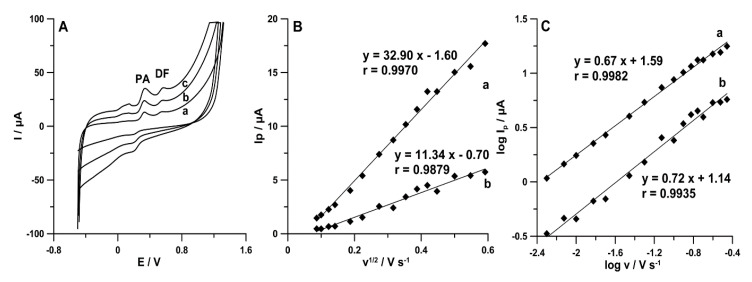
(**A**) CV curves recorded in the 0.15 mol L^−1^ acetate buffer solution of pH 4.0 containing 1.0 × 10^−4^ mol L^−1^ PA and 1.0 × 10^−6^ mol L^−1^ DF at *v* equal to (a) 50, (b) 100, and (c) 175 mV s^−1^. The dependence between (**B**) *Ip* and *v*^1/2^ and (**C**) log*Ip* and log*v* for PA (a) and DF (b).

The electrochemical responses of PA (1.0 × 10^−4^ mol L^−1^) and DF (1.0 × 10^−6^ mol L^−1^) at the SPCE/MWCNTs-COOH in the 0.15 mol L^−1^ acetate buffer solution of pH 4.0 were characterized by the CV technique. The scan rate was changed in the range of 5–350 mV s^−1^. On the basis of the obtained results (Figure 6A), it can be said that both PA and DF are irreversibly oxidized, giving rise to oxidation peaks at potentials around 330 and 550 mV, respectively, when the sweep was initiated in the positive direction. As can be seen, the oxidation peak potential of PA and DF shifted toward more positive values with the increasing scan rate. This confirms that PA and DF are irreversibly oxidized. Other peaks at less positive potentials are related to the formation of electrochemically active oxidation products of DF [4].

As can be seen in Figure 6B, the linear relationships between the PA and DF peak current (*Ip*) and the square root of scan rate (*v*^1/2^) indicated that the oxidation processes of PA (r = 0.9970) and DF (r = 0.9879) are controlled by diffusion at the SPCE/MWCNTs-COOH. However, the curve slopes of 0.67 (for PA) and 0.72 (for DF) observed in the plot of log*Ip* versus log*v* (Figure 6C) indicate that these processes are not purely diffusion- or adsorption-controlled [24]. Therefore, in the next step of the experiments, the effect of accumulation potential (*E_acc._*) was tested.

The effects of *E_acc._* at the SPCE/MWCNTs-COOH surface were studied in the mixed solution of PA (1.0 × 10^−6^ mol L^−1^) and DF (1.0 × 10^−8^ mol L^−1^). Keeping the accumulation time (*t_acc._*) as 60 s, the dependence of stripping peak current on *E_acc._* was evaluated over the potential range of 0.25 to −1.25 V. The peak current of PA and DF reached maximum at *E_acc._* of −0.25 V. This value of potential was chosen for further experiments. However, the constant value of potential was changed to pulsed potential accumulation.

**Figure 7:**
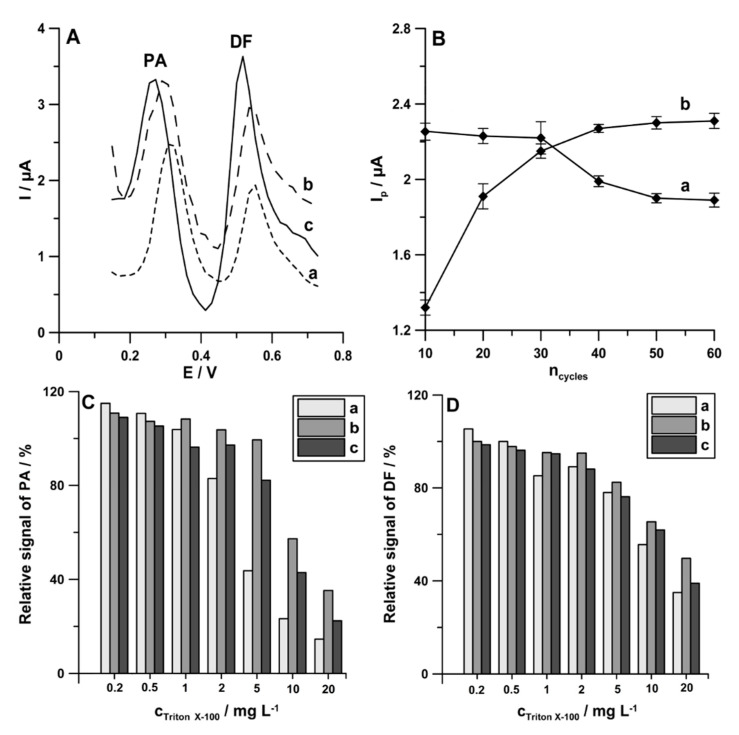
(**A**) DPAdSV curves registered in the solution containing PA (1.0 × 10^−6^ mol L^−1^) and DF (1.0 × 10^−8^ mol L^−1^) using the following: (a) accumulation potential of −0.25 V for 60 s; (b) a 1 s accumulation period at a potential of potential of −0.25 V and the anodic pulse with the differential-pulse scan from −0.25 to 0.1 V (*n_cycles_* = 60); (c) a 1 s accumulation period at a potential of 0.1 V and a 1 s accumulation period at a potential of −0.25 V (*n_cycles_* = 60). (**B**) Effect of *n_cycles_* on 1.0 × 10^−6^ mol L^−1^ PA (a) and 1.0 × 10^−8^ mol L^−1^ DF (b) current responses. Relative signals of 1.0 × 10^−6^ mol L^−1^ PA (**C**) and 1.0 × 10^−8^ mol L^−1^ DF (**D**) in the presence of increasing concentration of Triton X-100 with the parameters described in Figure 6A (curve c).

In voltammetric procedures, even a low concentration of surface active substances can foul and passify the electrode, causing a decrease or total decay of the analytical signal. UV irradiation or microwave heating before determination are recommended for elimination of this type of interference. However, such a process makes the procedures lengthy, complicated, and more expensive; requires additional apparatus; and cannot be used in field analysis. The literature also lists different simple and cheap ways for solving the problem with the organic matrix of natural water samples, namely application of potential pulses for accumulation. In addition, this way for the minimization of interferences can be applied outside laboratories. The potential of cathode pulses was chosen in a way that made it represent the maximum adsorption of the determined element and the potential of anode pulses to desorb the interfering surfactants [25,26]. Therefore, the procedure consisting of a 1 s accumulation period at a potential of 0.1 V (the anodic pulse) and a 1 s accumulation period at a potential of −0.25 V (the cathodic pulse) was proposed for simultaneous determination of PA (1.0 × 10^−6^ mol L^−1^) and DF (1.0 × 10^−8^ mol L^−1^). The differential-pulse scans from −0.25 to −0.254 V with an amplitude (*A*) of 150 mV, a modulation time (*t_m_*) of 20 ms, and a scan rate (*ν*) of 150 mV s^−1^ were recorded after 59 accumulation cycles. In the last cycle, the differential-pulse scan from −0.25 to 1.5 V was recorded with the parameters described above. Additionally, the procedure with a constant value of accumulation potential of −0.25 V for 60 s as well as the procedure consisting of a 1 s accumulation period at a potential of −0.25 V (the cathodic pulse) and the anodic pulse with the differential-pulse scan from −0.25 to 0.1 V (*n_cycles_* = 60) were applied. As can be seen in Figure 7A, the application procedure with pulsed potential accumulation (60-times pulses of 0.1 V for 1 s and −0.25 V for 1 s) improves both PA and DF analytical signals. To reduce the analysis time, the effect of number of cycles (*n_cycles_*) on the peak current of PA (1.0 × 10^−6^ mol L^−1^) and DF (1.0 × 10^−8^ mol L^−1^) was studied. Figure 7B shows the obtained results. For further experiments, the number of cycles was reduced to 30, as a compromise between the decrease in PA peak current and the increase in DF peak current.

According to the literature data, natural waters contain surfactants with the surface active effect similar to the effect induced by 0.2 to 2 ppm Triton X-100 [26]. Therefore, the effect of the use of pulsed potential accumulation of PA (1.0 × 10^−6^ mol L^−1^) and DF (1.0 × 10^−8^ mol L^−1^) on the minimization of interferences from surfactants was studied on the example of Triton X-100. As can be seen in Figure 7C,D, the application procedures with pulsed potential accumulation (b and c bars), compared with the application of a constant value of accumulation potential (a bars), contribute to the minimization of interferences from Triton X-100, particularly with regard to PA at a concentration of 2 ppm and upwards.

In summary, it can be stated that, in order to improve PA and DF analytical signals, as well as to minimize interferences from surfactants, pulsed potential accumulation can be applied. To our knowledge, this is the first time these two goals were achieved using pulsed potential accumulation. For further experiments, as a compromise between peak current and minimizing interference, the procedure consisting of a 1 s accumulation period at a potential of 0.1 V and a 1 s accumulation period at a potential of −0.25 V (*n_cycles_* = 30) was applied for the simultaneous determination of PA and DF.

It should be mentioned that the signals of PA and DF in the presence of other than Triton X-100 interferences found in environmental water samples were also studied. The tolerance limit was defined as the concentration that gives an error of ≤10% in the determination of 1.0 × 10^−6^ mol L^−1^ PA and 1.0 × 10^−8^ mol L^−1^ DF. It was noticed that uric acid (50-fold excess), urea (50-fold excess), ascorbic acid (100-fold excess), glucose (100-fold excess), dopamine (2-fold excess), Cu^2+^ (10-fold excess), Fe^3+^ (50-fold excess), Cd^2+^ (10-fold excess), Mo^6+^ (100-fold excess), Ni^2+^ (500-fold excess), Pb^2+^ (100-fold excess), Zn^2+^ (500-fold excess), Sb^3+^ (100-fold excess), V^5+^ (50-fold excess), K^+^ (100-fold excess), Na^+^ (100-fold excess), Cl^−^ (100-fold excess), SO_4_^2−^ (50-fold excess), PO_4_^3−^ (500-fold excess), and NO_3_^−^ (5000-fold excess) have a negligible effect on the assay of PA. Moreover, uric acid (5000-fold excess), urea (5000-fold excess), ascorbic acid (1000-fold excess), glucose (1000-fold excess), dopamine (1000-fold excess), Cu^2+^ (1000-fold excess), Fe^3+^ (5000-fold excess), Cd^2+^ (1000-fold excess), Mo^6+^ (5000-fold excess), Ni^2+^ (1000-fold excess), Pb^2+^ (5000-fold excess), Zn^2+^ (5000-fold excess), Sb^3+^ (10000-fold excess), V^5+^ (1000-fold excess), K^+^ (5000-fold excess), Na^+^ (5000-fold excess), Cl^−^ (5000-fold excess), SO_4_^2−^ (1000-fold excess), PO_4_^3−^ (50000-fold excess), and NO_3_^−^ (5000-fold excess) have a negligible effect on the assay of DF.

**Figure 8:**
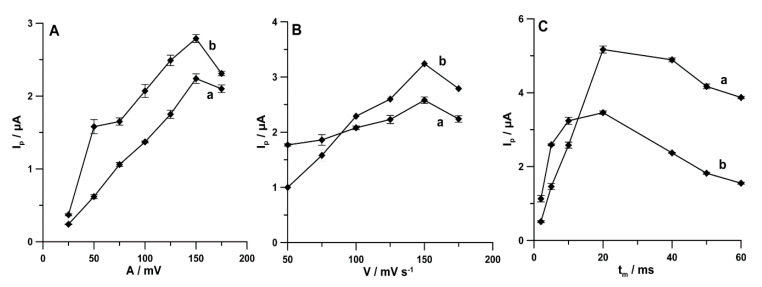
Effect of (**A**) *A* (25–170 mV), (**B**) *ν* (50–175 mV s^−1^), and (**C**) *t_m_* (2–60 ms) on PA (1.0 × 10^−6^ mol L^−1^) and DF (1.0 × 10^−8^ mol L^−1^). The DPAdSV parameters are as follows: *E_acc._* of 0.1 for 1 s and −0.25 V for 1 s, *n_cycles_* = 30, *ν* of 175 mV s^−1^, and *t_m_* of 10 ms (**A**); *A* of 150 mV and *t_m_* of 10 ms (**B**); and *A* of 150 mV and *ν* of 150 mV s^−1^ (**C**).

Furthermore, the influence of *A* on the analytical signals of PA (1.0 × 10^−6^ mol L^−1^) and DF (1.0 × 10^−8^ mol L^−1^) was examined from 25 to 175 mV (*ν* of 175 mV s^−1^ and *t_m_* of 10 ms). The highest peaks of both analytes were registered at *A* of 150 mV (Figure 8A). Next, the influence of *ν* (50–175 mV s^−1^) on the PA and DF peak current (*A* of 150 mV and *t_m_* of 10 ms) was tested. As can be seen in Figure 8B, the maximum values of PA and DF peak current were achieved at *ν* of 150 mV s^−1^. Additionally, *t_m_* was tested in the range of 2–60 ms (*A* of 150 mV and *ν* of 150 mV s^−1^). The highest signals of PA and DF were obtained for *t_m_* of 20 ms (Figure 8C).

### 3.4. The Linear Ranges, Limit of Detection (LOD), and Limit of Quantification (LOQ)

**Figure 9:**
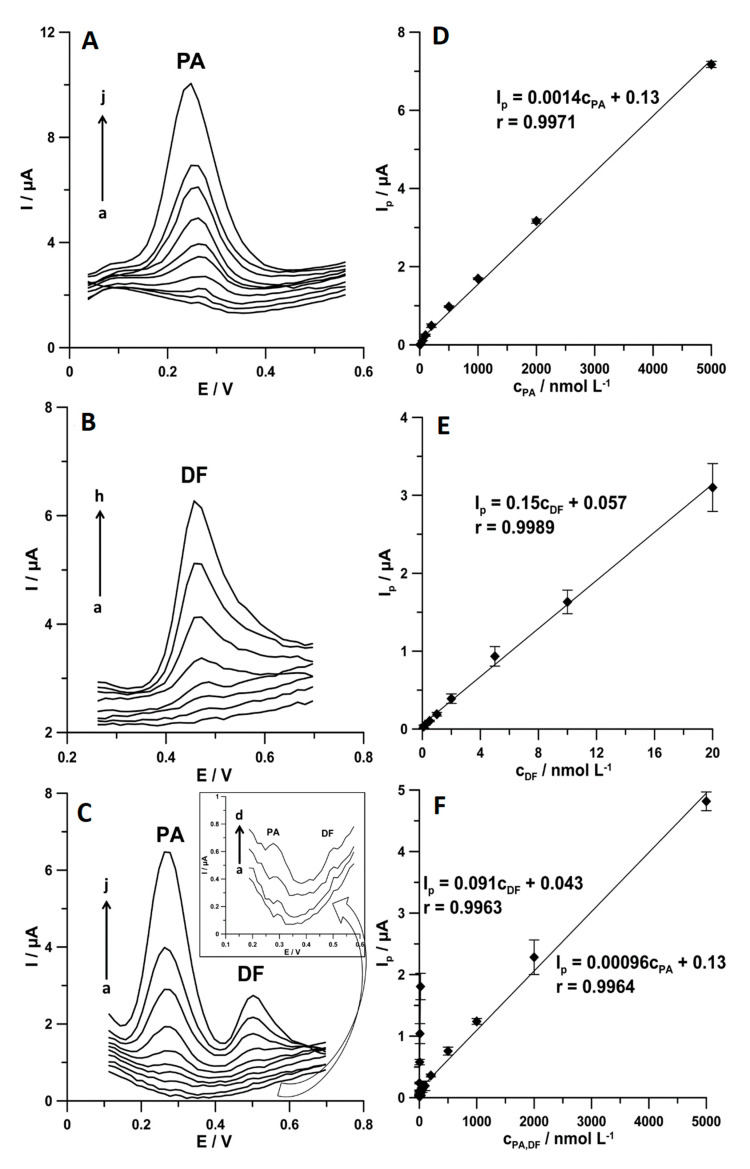
DPAdSV curves registered at the SPCE/MWCNTs-COOH in 0.15 mol L^−1^ the acetate buffer solution of pH 4.0 ± 0.1 containing increasing concentrations of the following: (**A**) PA (a–j, 5.0–5000.0 nmol L^−1^), (**B**) DF (a–h, 0.1–20.0 nmol L^−1^), and (**C**) PA (a–j, 5.0–5000.0 nmol L^−1^ and DF (a–h, 0.1–20.0 nmol L^−1^). Calibration graph of (**D**) PA, (**E**) DF, and (**F**) PA and DF. The DPAdSV parameters are as follows: *E_acc._* of 0.1 for 1 s and −0.25 V for 1 s, *n_cycles_* = 30, *A* of 150 mV, *ν* of 150 mV s^−1^, and *t_m_* of 20 ms.

Figure 9 shows the DPAdSV curves and linear ranges of calibration plots obtained under optimized conditions during individual determination of PA and DF as well as during simultaneous determination of these compounds. The results are summarized in Table 1. The limits of detection (LOD) and quantification (LOQ) obtained during simultaneous determination of PA and DF are 1.44 and 4.80 nmol L^−1^ and 0.030 and 0.1 nmol L^−1^, respectively. These results demonstrate that the SPCE/MWCNTs-COOH can be applied to environmental water samples analysis in which PA and DF concentrations are in the range of 10^−9^–10^−8^ and 10^−11^–10^−8^ mol L^−1^, respectively [7,8]. Table 2 shows the comparison techniques used for the simultaneous determination of PA and DF. It can be summarized that the DPAdSV with SPCE/MWCNTs-COOH allows the lowest LOD value to be obtained compared with all other electrochemical sensors and techniques [9,10,11,12,13,14,15,16,17,18,19].

### 3.5. Precision and Reproducibility

The intra-day and inter-day precision were examined by measuring the stopping responses of 1.0 × 10^−6^ mol L^−1^ PA and 1.0 × 10^−8^ mol L^−1^ DF with 10 replicates on 1 day and 3 replicates on 5 days, respectively. The relative standard deviations (RSDs) are 3.7% (n = 10) and 5.1% (n = 15) for PA, and 5.3% (n = 10) and 6.2% (n = 15) for DF, indicating satisfactory precision of the signals at the SPCE/MWCNTs-COOH. The reproducibility was evaluated by recording DPAdSV curves in the solution of 1.0 × 10^−6^ mol L^−1^ PA and 1.0 × 10^−8^ mol L^−1^ DF using three electrodes. The RSD was calculated as 4.9% (n = 9, for PA) and 5.2% (n = 9, for DF), approving the acceptable reproducibility of the sensor.

### 3.6. Analytical Applications

**Figure 10:**
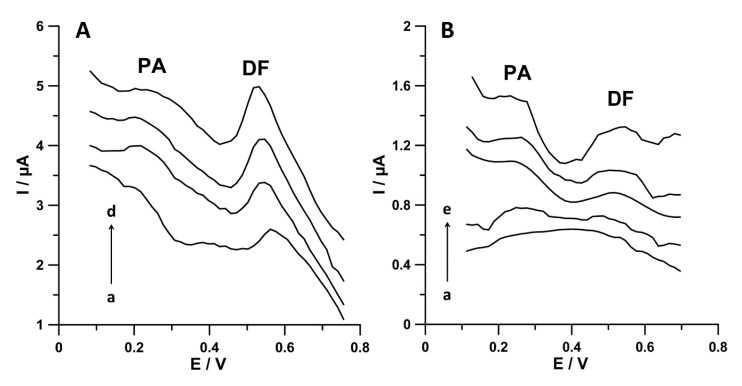
DPAdSV curves registered at the SPCE/MWCNTs-COOH during PA and DF simultaneous analysis in (**A**) wastewater samples purified in a sewage treatment plant (a) 5 mL of sample, (b) as (a) + 5.0 nmol L^−1^ PA and 0.5 nmol L^−1^ DF, (c) as (a) + 10.0 nmol L^−1^ PA and 1.0 nmol L^−1^ DF, and (d) as (a) + 15.0 nmol L^−1^ PA and 1.5 nmol L^−1^ DF; (**B**) Bystrzyca river water sample: (a) 5.0 mL of sample, (b) as (a) + 5.0 nmol L^−1^ PA and 0.5 nmol L^−1^ DF, (c) as (a) + 10.0 nmol L^−1^ PA and 1.0 nmol L^−1^ DF, (d) as (a) + 15.0 nmol L^−1^ PA and 1.5 nmol L^−1^ DF, and (e) as (a) + 20.0 nmol L^−1^ PA and 2.0 nmol L^−1^ DF. The DPAdSV parameters are as follows: *E_acc._* of 0.1 for 1 s and −0.25 V for 1 s, *n_cycles_* = 30, *A* of 150 mV, *ν* of 150 mV s^−1^, and *t_m_* of 20 ms.

Finally, the practical application of the proposed voltammetric procedure using SPCE/MWCNTs-COOH was illustrated by simultaneous determination of PA and DF in Bystrzyca river samples and wastewater samples purified in a sewage treatment plant. The voltammetric results were compared to those obtained by chromatographic method (HPLC/PAD) and summarised in Table 3. Figure 10 shows the DPAdSV curves obtained during simultaneous determination of PA and DF in the analysed samples. The results achieved by the voltammetric method show satisfactory agreement with those obtained by HPLC/PAD (the relative errors are in the range of 1.1–6.7%). In order to test the accuracy of the voltammetric procedure, the samples were spiked with standard solutions of PA and DF. The recovery values are between 96.5% and 104.8%, which corresponds to the satisfactory degree of accuracy.

It needs to be highlighted that only the voltammetric procedure using the SPCE/MWCNTs-COOH allows simultaneous determination of PA and DF at concentrations of 24.3 ± 0.5 nmol L^−1^ and 3.7 ± 0.7 nmol L^−1^, respectively, in wastewater samples purified in a sewage treatment plant. These results show that, after leaving the sewage treatment plant, the wastewater still contains PA and DF, which then end up in the natural environment. The concentrations of PA and DF in Bystrzyca river samples below the limit of detection of the DPAdSV technique confirm the dilution of analytes.

## 4. Conclusions

For the first time, in this study, carboxyl functionalized multiwalled carbon nanotubes modified screen-printed carbon electrode (SPCE/MWCNTs-COOH) was introduced for the simultaneous, direct analysis of low concentrations of paracetamol (PA) and diclofenac (DF). Moreover, for the first time, pulsed potential accumulation was used in order to improve PA and DF analytical signals and to minimize interferences from surfactants.

In this work, already published results regarding the electrochemical properties of SPCE and SPCE/MWCNTs-COOH [4] were compared with these obtained for SPCE/CNFs. The results show that the SPCE/MWCNTs-COOH has a greater number of active centers than the unmodified SPCE and the SPCE/CNFs, which explain the enhancement of PA and DF signals in relation to the SPCE, and the DF signal in relation to the SPCE/CNFs. The SPCE/MWCNTs-COOH was recommended for simultaneous analysis of PA and DF, but the SPCE/CNFs for the individual analysis of PA. Moreover, the electrochemical responses of PA and DF at the SPCE/MWCNTs-COOH in the 0.15 mol L^−1^ acetate buffer solution (pH 4.0) were characterized by the CV technique. The obtained results indicated that the oxidation processes of PA and DF at the SPCE/MWCNTs-COOH are not purely diffusion- or adsorption-controlled.

Moreover, only the proposed voltammetric procedure using the SPCE/MWCNTs-COOH allows simultaneous determination of PA and DF at concentrations of 24.3 ± 0.5 nmol L^−1^ and 3.7 ± 0.7 nmol L^−1^, respectively, in wastewater samples purified in a sewage treatment plant. These results show that, after leaving the sewage treatment plant, the wastewater still contains PA and DF, which then end up in the natural environment. It should be clearly emphasized that the samples were directly analysed without performing any special sample pretreatment procedure.

The proposed voltammetric procedure has the advantages of being much more sensitive, less time-consuming, and less expensive than HPLC. Moreover, the analysis of water samples can be carried out in the laboratory and at the place of sampling.

## Figures and Tables

**Table 1:** Characteristics of calibration plots of paracetamol (PA) and diclofenac (DF) attained at the commercially available carboxyl functionalized multiwalled carbon nanotubes modified screen-printed carbon electrodes (SPCE/MWCNTs-COOH). LOD, limit of detection; LOQ, limit of quantification.

Parameter	PA	DF	PA and DF
Linear range [nmol L^−1^]	5.0–5000.0	0.1–20.0	5.0–5000.0 (PA) 0.1–20.0 (DF)
Slope (b) ± SD_b_ (n = 3) [µA/nmol L^−1^]	0.0014 ± 0.000010	0.15 ± 0.025	0.00096 ± 0.000044 (PA) 0.091 ± 0.012 (DF)
Intercept (a) ± SD_a_ (n = 3) [µA]	0.13 ± 0.00062	0.057 ± 0.00079	0.13 ± 0.00046 (PA) 0.043 ± 0.00092 (DF)
Correlation coefficient (r)	0.9971	0.9989	0.9964 (PA) 0.9963 (DF)
LOD [nmol L^−1^]	1.34	0.015	1.44 (PA) 0.030(DF)
LOQ [nmol L^−1^]	4.47	0.051	4.80 (PA) 0.10 (DF)

*LOD = 3SD_a/b_ and LOQ = 3SD_a/b_* [27].

**Table 2:** Comparison of techniques for simultaneous analysis of PA and DF.

Technique	Analyte	Linear Range [mol L^−1^]	Detection Limit [mol L^−1^]	Application	Ref.
RP-HPLC	PA DF	1.1 × 10^−6^–6.6 × 10^−5^ 6.3 × 10^−8^–3.1 × 10^−5^	-	Pharmaceutical, Human serum	[9]
RP-HPLC	PA DF	3.3 × 10^−4^–9.9 × 10^−4^ 1.6 × 10^−5^–4.7 × 10^−5^	1.3 × 10^−8^ 7.9 × 10^−8^	Pharmaceutical	[10]
HPLC	PA DF	6.6 × 10^−9^–6.6 × 10^−7^ 3.1 × 10^−9^–3.1 × 10^−7^	4.4 × 10^−8^ 9.7 × 10^−10^	Wastewater samples	[11]
RP-HPLC	PA DF	6.6 × 10^−6^–2.0 × 10^−4^ 3.1 × 10^−6^–1.0 × 10^−4^	2.2 × 10^−5^ 1.1 × 10^−6^	Pharmaceutical	[12]
GC-MS	PA DF	1.1 × 10^−7^–6.6 × 10^−5^ 2.8 × 10^−8^–3.1 × 10^−5^	-	Sea water, Wastewater	[13]
Spectrophotometric	PA DF	6.6 × 10^−6^–2.0 × 10^−4^ 1.6 × 10^−6^–1.0 × 10^−4^	1.2 × 10^−6^ 1.6 × 10^−7^	Pharmaceutical	[14]
Electrophoresis	PA DF	3.3 × 10^−5^–8.3 × 10^−4^ 3.1 × 10^−6^–3.9 × 10^−4^	6.6 × 10^−6^ 1.6 × 10^−6^	Pharmaceutical, Human serum	[15]
Electrophoresis	PA DF	3.3 × 10^−5^–1.7 × 10^−3^ 3.1 × 10^−6^–3.9 × 10^−4^	6.6 × 10^−6^ 1.6 × 10^−6^	Pharmaceutical, Urine sample	[16]
4-PP/GCE	PA DF	1.9 × 10^−6^–1.7 × 10^−4^ 3.7 × 10^−7^–5.2 × 10^−5^	-	Drug delivery system	[17]
PDDA/GR/GCE	PA DF	3.0 × 10^−6^–2.0 × 10^−4^ 1.0 × 10^−5^–1.0 × 10^−4^	2.2 × 10^−7^ 6.1 × 10^−7^	Pharmaceutical, Lake water	[18]
AuNPs-GR/PAG/GCE	PA DF	5.0 × 10^−7^–5.0 × 10^−5^ 5.0 × 10^−7^–4.0 × 10^−5^	4.0 × 10^−8^ 8.0 × 10^−8^	Human serum	[19]
SPCE/MWCNTs-COOH	PA DF	5.0 × 10^−9^–5.0 × 10^−6^ 1.0 × 10^−10^–2.0 × 10^−8^	1.4 × 10^−9^ 3.0 × 10^−11^	River water, Wastewater	This work

4-PP/GCE—4-phosphatephenyl modified glassy carbon electrode; PDDA/GR/GCE—poly(diallyldimethylammonium chloride) functionalized graphene modified glassy carbon electrode; AuNPs/GR/PAG/GCE—poly(L-Arginine)/Au-graphene nanocomposite film deposited on a glassy carbon electrode.

**Table 3:** Results of simultaneous determination of PA and DF in environmental water samples. DPAdSV, differential-pulse adsorptive stripping voltammetric.

**Sample**	**PA Concentration [nmol L^−1^] ± SD (n = 3)**			Recovery * [%]	Relative Error ** [%]
	**Added**	**Found DPAdSV**	**Found HPLC/PAD**		
Bystrzca	0	<LOD	<LOD	-	-
river	5.0	5.09 ± 0.044	<LOD	101.8	-
	500.0	505.0 ± 4.0	514.0 ± 6.5	101.0	1.8
Waste-	0	24.3 ± 0.5	25.4 ± 6.0	-	4.3
water	5.0	29.2 ± 5.5	31.3 ± 2.7	99.7	6.7
	500.0	523.0 ± 9.0	529.0 ± 8.6	99.8	1.1
	**DF Concentration [nmol L^−1^] ± SD (n = 3)**			Recovery * [%]	Relative Error ** [%]
	**Added**	**Found DPAdSV**	**Found HPLC/PAD**		
Bystrzca	0	<LOD	<LOD	-	-
river	0.5	0.51 ± 0.0066	<LOD	102.0	-
	50.0	50.5 ± 0.4	49.6 ± 0.8	101.0	1.8
Waste-	0	3.7 ± 0.7	<LOD	-	-
water	0.5	4.4 ± 0.6	<LOD	104.8	-
	50.0	51.8 ± 0.7	49.7 ± 1.1	96.5	4.2

* Recovery [%] = (Found DPAdSV × 100)/Added; ** Relative error [%] = ((ǀFound HPLC/PAD–Found DPAdSVǀ)/Found HPLC/PAD) × 100.
